# LED-FISH: Fluorescence microscopy based on light emitting diodes for the molecular analysis of Her-2/neu oncogene amplification

**DOI:** 10.1186/1746-1596-3-49

**Published:** 2008-12-16

**Authors:** Dagmar S Lang, Tobias Zeiser, Holger Schultz, Florian Stellmacher, Ekkehard Vollmer, Peter Zabel, Torsten Goldmann

**Affiliations:** 1 Clinical and Experimental Pathology, Research Center Borstel, Parkallee 3, D-23845 Borstel, Germany; 2 Paracelsus Klinik Henstedt-Ulzburg, Department of Gynecology and Obstetrics, Schützenstrasse 55, D-23843 Bad Oldesloe, Germany; 3 Medical University Hospitall III Lübeck/Medical Clinic, Research Center Borstel, Parkallee 35, D-23845 Borstel, Germany/Ratzeburger Allee 160, D-23562 Lübeck, Germany

## Abstract

Light emitting diodes (LED), which are available as small monochromatic light sources with characteristic features such as maximum illumination power combined with minimum energy consumption and extremely long lifespan have already proved as a highly potential low-cost alternative for specific diagnostic applications in clinical medicine such as tuberculosis fluorescence microscopy. Likewise, the most reliable evaluation of Her-2/neu (c-erbB2) gene amplification, which has been established in the last few years for routine diagnosis in clinical pathology as determinant towards Herceptin-based treatment of patients with breast cancer, is based on fluorescence *in situ *hybridization (FISH) and corresponding high priced fluorescence equipment. In order to test the possibility to utilize the advantages of low-cost LED technology on FISH analysis of c-erbB2 gene expression for routine diagnostic purposes, the applicability of a standard bright field Carl Zeiss Axiostar Plus microscope equipped with a Fraen AFTER* LED Fluorescence Microscope Kit for the detection of Her-2/neu gene signals was compared to an advanced Nikon Eclipse 80i fluorescence microscope in combination with a conventional 100W mercury vapor lamp. Both microscopes were fitted with the same Quicam FAST CCD digital camera to unequivocally compare the quality of the captured images. C-erbB2 gene expression was analyzed in 30 different human tissue samples of primary invasive breast cancer, following formalin fixation and subsequent paraffin-embedding. The Her2/neu gene signals (green) were identifiable in the tumor cells in all cases and images of equal quality were captured under almost identical conditions by 480 nm (blue) LED module equipped standard Axiostar microscope as compared to conventional fluorescence microscopy. In this first attempt, these monochromatic LED elements proved in principle to be suitable for the detection of Her-2/neu gene expression by FISH. Thus, our own experiences emphasize the high potential of this technology to provide a serious alternative to conventional fluorescence microscopy in routine pathology; representing a sustainable technological progress, this low-cost technology will clearly give direction also to the growing field of molecular pathology.

* AFTER = Amplified Fluorescence by transmitted Excitation of Radiation

## Findings

Light Emitting Diodes (LED) are characterized by low cost, effective energy consumption and extremely long lifespan when compared to conventional light sources [1]. Both the small size and the lack of heat development by LED elements also contribute to these major advantages above conventional lighting technology that altogether imply considerable economic cost reductions. As a consequence, there has been a fast propagation of LED elements as important constituents in many different branches such as automobile industry, household or camping [2,3]. More recently, the availability of a variety of small monochromatic LED modules efficiently emitting the spectrum in a single desired bandwidth has stimulated the interest of clinical researchers to utilize this low-cost technology for advanced fluorescence microscopy in diagnostic research [4]. Since LED based light sources operate without increasing their temperature, common safety problems related to considerable heat production by conventional high pressure mercury lamps are completely avoided. Most recently, LED modules attached to standard light microscopes have been successfully applied in fluorescence-based screening of tuberculosis, pointing to the considerable reduction of related costs combined with increased safety and, as a consequence, to the potential for low-income countries to perform such advanced diagnostics of this disease in the near future [5,6]. Likewise, the increasing application of highly sensitive but also expensive molecular methods in clinical pathology such as the analysis of altered gene expression patterns for cancer diagnosis, has led to a general demand for reducing costs in routine processes. In the last few years, the evaluation of Her-2/neu status has considerably gained clinical importance related to the selection of patients with breast cancer, who will benefit most from a novel targeted therapy based on Herceptin, a humanized monoclonal antibody directed against this protein [7,8]. Thus, overexpression of Her-2/neu protein represents one of only few available predictive markers for an individualized and more efficient treatment regimen in this type of cancer [9]. Since the enhancement of protein levels is primarily correlated to the amplification of the corresponding gene c-erbB2, fluorescence *in situ *hybridization (FISH) has been established for the determination of Her-2/neu gene. FISH is characterized by excellent sensitivity (96.5%) and specificity (100%) [10]. Thus, this diagnostic assay has been introduced in routine clinical pathology, despite the considerable costs involving expensive reagents and the need to purchase high priced fluorescent equipment before deciding the further steps in the comparably much more expensive treatment with all its possible therapeutic side effects 1.

**Figure 1:**
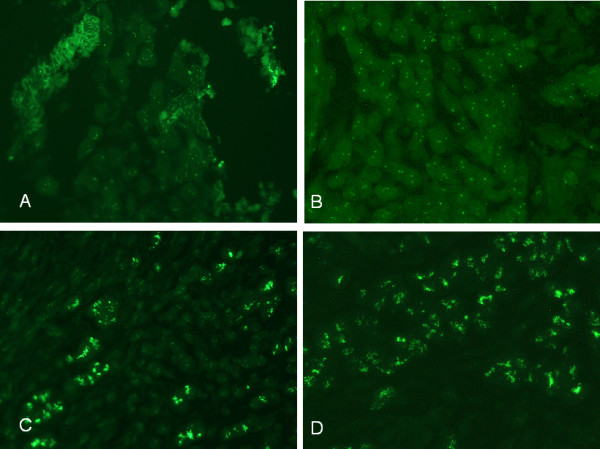
**Detection of Her-2/neu gene expression in human breast cancer tissue by FISH analysis**. Two exemplary tissues of breast cancer exhibiting no amplification (**A, B**) or strong amplification of Her-2/neu gene (**C, D**) are shown (400× magnification). ZyGreen labeled Her-2/neu gene signals captured by advanced Nikon fluorescent microscope in combination with conventional 100W mercury vapor lamp (**A, C**) are directly compared with those captured by standard bright field Carl Zeiss Axiostar Plus microscope equipped with a Fraen AFTER LED Fluorescence Microscope Kit (**B, D**). Cerb-B2 gene expression was detected by a 480 nm Fluorescence light cassette combined with a 510 nm long pass emission filter mounted to the Axiostar microscope. Equal quality of the captured pictures was achieved by integration times between 1 and 3 seconds (A, C, 100W mercury vapor) and 6 and 10 seconds (B, D, LED).

For this reason, the use of small monochromatic LED modules as the required light source for routinely performed FISH analysis of Her-2/neu status appeared to us as a promising alternative to eventually replace the short-lived and expensive conventional mercury vapor lamp. For this purpose, a commercially available AFTER (Amplified Fluorescence by transmitted Excitation of Radiation) LED Fluorescence Microscope Kit (Lab Vision, Fremont, USA) was mounted to a standard Zeiss Axiostar Plus transmitted light microscope (Medac, Wedel, Germany), providing a simple adaptation of a fluorescence microscope. Determination of ZyGreen c-erb-B2 gene was performed by attaching a 480 nm Fraen fluorescence light cassette to the Axiostar Plus in combination with a LP 510 nm long pass filter. For comparison reasons, the Zeiss microscope was also fitted with a Quicam FAST CCD digital camera, which is normally attached to the Nikon Eclipse 80i fluorescence microscope for routine determination of cerb-B2 gene amplification. Her-2/neu gene expression was documented at 400× magnification. A total of 30 tissue samples from patients with primary invasive breast carcinoma were analyzed by FISH. After mastectomy, the specimens were fixed by formalin and subsequently paraffin-embedded. Optimal comparability among all samples was achieved by producing a tissue micro array (TMA), as previously described [11]. FISH was performed, using ZytoLight Spec Her-2 Color Probe (ZytoVision GmbH, Bremerhaven, Germany). Briefly, a 4 μm thick section of the TMA was deparaffinized with xylene, dehydrated and pre-treated with enzyme and heat, according to the manufacturer's instructions. After addition of 10 μl SPEC HER2 Color probe, denaturation was carried out at 75°C for 10 min, followed by hybridization overnight at 37°C. Post hybridization washing, subsequent dehydration in ethanol and counterstaining with DAPI (4,6-diaminido-2-phenylindole dihydrochloride)/antifade-solution was performed, as specified. The slides were kept in the dark at 4°C until evaluation.

The age of the patients ranged between 44 years and 84 years (median age 61 y) with 77% of the women being in the postmenopausal phase. The majority of the breast cancer tissues (86%) were related histologically to the invasive ductal type of tumor. Amplification of cerb-B2 gene was detected by FISH in 36% of the specimens. Brightness of the Her-2/neu gene signals was always sufficient using the LED equipped Axiostar Plus microscope and corresponding photographs of high quality were captured by extended integration times between 3 and 10 seconds.

To our knowledge, this is the first attempt to use LED modules instead of a 100 W mercury vapor lamp as light source to perform FISH analysis of clinically relevant Her-2/neu gene expression for routine pathology. Up to date, the introduction of LED technology in diagnostic research has been very successful as recently demonstrated for the fluorescence based screening of tuberculosis [5,6,12]. Likewise, in our studies the standard light transmission microscope Axiostar Plus became suitable for FISH analysis by simply attaching a commercially available adaptation kit for fluorescence microscopy. The appropriate combination of a particular LED module and the corresponding long pass emission filter was sufficient to replace the advanced Nikon fluorescence microscope. Moreover, the lack of heat production by the LED light sources completely avoided common safety problems related to conventional mercury vapor lamps. The extension of the integration time was the only major modification of the otherwise identical conditions to capture pictures of equal quality as compared to the routinely used high priced fluorescence equipment. Integration times less than 3 times shorter to be sufficient for documentation, as demonstrated for the 100 W mercury lamp, still emphasize the need for LED modules with further increased illumination power. Moreover, the development of LED light sources with corresponding long pass emission filters that are suitable for the detection of ZyOrange labeled probes would enable the combined analysis of both cerb-B2 gene and corresponding chromosome 17 as the most reliable determination of the Her-2/neu status in patients with breast cancer. In addition, the possibility to simply switch between different LED modules instead of the necessity to exchange the whole elements would considerably simplify the applicability of LED elements, since fluorescence based assays such as FISH using commercially available dual color labeled kits are increasingly introduced into clinical research. In summary, although there is still need for some further developments, our own experiences emphasize the high potential of these monochromatic LED elements with all their characteristic features to provide a serious alternative to conventional advanced fluorescence microscopy in routine pathology. Without dispute this low-cost technology has initiated a sustainable technological progress giving direction also to the growing field of molecular pathology.

## Competing interests

The authors declare that they have no competing interests.

## Authors' contributions

DSL carried out the FISH analyses and drafted the manuscript. TZ was responsible for the surgical part and clinical data. PZ provided the technological capabilities. HS and EV were responsible for the histopathological aspects. TG conceived of the study and was involved in drafting the manuscript. All authors have read and approved the final manuscript.
